# Age-specific profiles of suicide warning signs among Korean suicide decedents, 2013–2020

**DOI:** 10.3389/fpubh.2026.1969435

**Published:** 2026-10-07

**Authors:** Junggeun Ahn, Jiu Kim

**Affiliations:** 1 College of Nursing, Jeju National University, Jeju, Republic of Korea; 2 Health and Nursing Research Institute, Jeju National University, Jeju, Republic of Korea; 3 College of Nursing, Dongguk University-WISE, Gyeongju, Republic of Korea

**Keywords:** age differences, suicide, suicide decedents, suicide prevention, warning signs

## Abstract

**Introduction:**

Suicide warning signs may provide important opportunities for early recognition and intervention, yet their presentation may vary across the life course. However, population-level evidence on age-specific patterns of suicide warning signs remains limited. This study examined age-group differences in the presence and types of suicide warning signs among suicide decedents in South Korea.

**Methods:**

We analyzed data from the Korean National Investigations of Suicide Deaths Through Police Records, 2013–2020. The analytic sample included 96,501 suicide decedents with valid information on age group and warning sign status. Participants were categorized as children/adolescents, young adults, middle-aged adults, or older adults. Chi-square tests were used to examine age-group differences in the presence of any warning sign, and binary logistic regression was conducted to assess age-group differences in any warning sign. Separate item-level logistic regression models were used for specific warning signs.

**Results:**

Overall, 92.9% of suicide decedents had at least one warning sign. The proportion was highest among older adults (95.1%) and lowest among children/adolescents (87.4%). Compared with older adults, all younger age groups had lower odds of having any recorded warning sign. The types of warning signs also differed by age. Verbal warning signs, particularly complaints of physical discomfort, were more characteristic of older adults, whereas emotional warning signs were more prominent among children/adolescents and young adults. Behavioral warning signs were more frequently observed among younger and middle-aged adults than among older adults.

**Conclusion:**

Suicide warning signs vary substantially across age groups, suggesting that a uniform approach to warning sign recognition may overlook age-specific patterns. These findings may inform future age-sensitive recognition strategies and improvements in suicide mortality surveillance.

## 1 Introduction

Suicide prevention requires attention not only to who is at risk, but also to how suicide risk becomes observable before death. Although psychiatric disorders, previous self-harm, substance misuse, and adverse social circumstances are well-established risk factors for suicide mortality, these factors do not always indicate when a suicidal crisis is becoming imminent (1, 2). In addition, many people who die by suicide have limited or no contact with mental health services before death, reducing the effectiveness of prevention strategies that depend primarily on clinical detection (3). These limitations highlight the need to examine warning signs that may be observed outside clinical settings, including by family members, peers, neighbors, community workers, and other people who encounter individuals in their everyday lives.

Suicide warning signs are conceptually different from distal risk factors. Risk factors indicate vulnerability over time, whereas warning signs refer to observable verbal, behavioral, or emotional changes that may suggest more immediate suicide risk (4). Such signs may include communication about death or suicide, marked changes in behavior, withdrawal from others, emotional instability, substance misuse, or other deviations from usual functioning. Because warning signs are closer to the suicidal crisis, they may provide practical opportunities for earlier recognition and timely intervention.

Despite their prevention relevance, warning signs have received less systematic attention than broader suicide risk factors. Prior research has shown that warning signs can be difficult to recognize because they may be indirect, context-dependent, or interpreted as ordinary distress rather than as indicators of suicide risk (5). Korean studies using psychological autopsy or police-record-based data have also shown that warning signs are frequently documented before suicide and may vary by timing, psychiatric history, and observability (6–8). These findings suggest that warning signs should not be treated simply as a single yes-or-no phenomenon; rather, their specific forms and meanings may differ depending on the characteristics and life context of the decedent.

Age is one of the key contexts that may shape how suicide warning signs are expressed and recognized. Across the life course, individuals differ in developmental tasks, social roles, health status, interpersonal relationships, communication patterns, and help-seeking behavior. These differences may influence whether suicide risk becomes visible through verbal expressions, behavioral changes, emotional symptoms, physical complaints, or disclosure of suicidal intent. Recent studies support this age-sensitive perspective. Research on Korean adolescents has shown that warning signs may be difficult to recognize when emotional distress is concealed or when outward school and peer functioning appears relatively preserved (8). Studies of adult and older adult suicide decedents have also found that suicidal intent disclosure and its correlates differ by age group, with age-related variation in the roles of relationship problems, health problems, suicide means, and other contextual factors (9, 10). Together, these findings indicate that suicide warning signs may be better understood as age-specific profiles rather than as uniform indicators that appear similarly across all decedents.

Korea is an important setting in which to examine age-specific warning sign profiles. Suicide remains a major public health concern in Korea, and recent OECD data indicate that Korea continues to report suicide rates above the OECD average (11). However, large-scale national evidence on how warning signs differ across age groups among Korean suicide decedents remains limited. Most previous work has focused on the overall presence of warning signs, specific clinical subgroups, or selected age groups (1, 12, 13). Less is known about how verbal, behavioral, and emotional warning signs are distributed across adolescents, young adults, middle-aged adults, and older adults within the same national dataset.

Therefore, this study examined age-specific profiles of suicide warning signs among Korean suicide decedents using data from The Korean National Investigations of Suicide Deaths Through Police Records from 2013 to 2020. Specifically, this study compared the presence of any warning sign across children/adolescents, young adults, middle-aged adults, and older adults, and examined age-group differences in specific verbal, behavioral, and emotional warning signs.

## 2 Methods

### 2.1 Study design and reporting framework

This study used a retrospective secondary data analysis design. The manuscript was prepared with reference to the Strengthening the Reporting of Observational Studies in Epidemiology (STROBE) recommendations for observational studies (14). The study examined age-specific profiles of suicide-related warning signs among Korean suicide decedents.

### 2.2 Data source and study population

Data were obtained from The Korean National Investigations of Suicide Deaths Through Police Records, a nationwide database based on police investigation records of deaths officially concluded as suicide in Korea. The database was coded by trained investigators at the Korea Foundation for Suicide Prevention, an agency under the Ministry of Health and Welfare. The present analysis used data from suicide decedents recorded between 2013 and 2020.

The original dataset included 102,922 suicide decedents. User-defined missing values were treated as missing in all analyses. No weighting, filtering, or split-file procedure was applied. Because missingness differed across variables, analyses were conducted using analysis-specific valid cases. The primary cross-tabulation of age group and the presence of any suicide warning sign included 96,501 valid cases; 6,421 cases were excluded from this analysis because age group or warning sign status was missing or coded as unknown. The primary logistic regression model including age group and sex included 96,487 valid cases; 14 additional cases were excluded because of missing sex. For item-level logistic regression models, analytic sample sizes varied by warning sign item because of variable-specific missingness.

### 2.3 Measures

The main independent variable was age group. Age was originally recorded in 5-year categories and recoded into four life-course groups: children/adolescents aged 19 years or younger, young adults aged 20–39 years, middle-aged adults aged 40–64 years, and older adults aged 65 years or older. These cutoffs were selected to distinguish minors and school-age youth, early and established adulthood, midlife adulthood, and later life. Because the source data were provided in 5-year age bands, the <=19 years group included preadolescent children as well as adolescents. In the logistic regression analyses, older adults served as the reference group.

The primary outcome was the presence of any suicide-related warning sign before death. Warning signs were conceptualized as observable verbal, behavioral, or emotional indicators that may suggest more immediate suicide risk, distinct from more distal suicide risk factors (4). Warning sign information was coded from police investigation reports that had been prepared based on sources such as statements from bereaved family members, acquaintances, and coworkers, as well as suicide notes when available. The Korea Foundation for Suicide Prevention provided standardized coding training to investigators, and investigators who completed this training coded the police investigation reports. The original warning sign variable was recoded into a binary variable, with cases coded as 1 if at least one warning sign was present and 0 if no warning sign was identified in the record-based coding framework. Thus, a code of absence indicates that the warning sign was coded as absent based on the available police investigation report, while still reflecting the inherent limits of retrospective record-based ascertainment.

Specific warning signs were examined in three domains: verbal, behavioral, and emotional warning signs. Verbal warning signs included statements about suicide, homicide, or death; complaints of physical discomfort; and self-deprecating statements. Behavioral warning signs included indifference to appearance management; self-harm, suicide attempts, or substance misuse; and apathy, interpersonal withdrawal, or loss of interest. Emotional warning signs included depressed mood, elevated mood, and anxiety. In the original coding framework, elevated mood referred to a state that included mood that was unusually elevated compared with the decedent’s usual state, emotional elevation, excitement, or agitation before death.

Each warning sign item was coded as present or absent within the standardized record-based coding framework. Because multiple warning signs could be recorded for the same decedent, the specific warning sign items were analyzed as non-mutually exclusive indicators. Sex was included as a covariate in the primary adjusted logistic regression model for the presence of any warning sign, with female decedents serving as the reference group.

### 2.4 Statistical analysis

All statistical analyses were conducted using SPSS. First, frequencies and percentages were calculated to describe the distribution of suicide warning signs by age group. The presence of any warning sign was summarized as a binary outcome, and the composition of verbal, behavioral, and emotional warning sign occurrences was described within each age group.

Second, chi-square tests were conducted to examine whether the presence of any warning sign differed across age groups. The primary cross-tabulation compared warning sign presence versus absence among children/adolescents, young adults, middle-aged adults, and older adults.

Third, binary logistic regression was performed to estimate age-group differences in the presence of any warning sign. The primary adjusted model included age group and sex as predictors. Odds ratios (ORs), 95% confidence intervals (CIs), and *p*-values were reported.

Finally, separate binary logistic regression models were conducted for each specific verbal, behavioral, and emotional warning sign item to examine age-group differences in specific warning signs. Older adults were used as the reference group in these models. These item-level models included age group only and were not adjusted for sex because their purpose was to describe age-specific warning sign profiles rather than to estimate sex-adjusted independent age effects for each item. Accordingly, they were interpreted as descriptive age-group comparisons rather than causal or fully adjusted estimates. Analytic sample sizes were 82,795–82,813 for verbal warning sign models, 76,912–76,918 for behavioral warning sign models, and 76,232–76,255 for emotional warning sign models. Together, the descriptive comparisons and item-level regression analyses were used to characterize age-specific profiles of suicide warning signs. Statistical significance was set at *p* < 0.05.

For descriptive analyses of warning sign domains, percentages were calculated based on warning sign occurrences rather than mutually exclusive person-level categories, because a single decedent could have more than one warning sign. Accordingly, these percentages describe the composition of warning sign occurrences within each age group and should not be interpreted as person-level prevalence of each domain.

### 2.5 Ethical considerations

This study used de-identified secondary data derived from police investigation records. The study was granted an exemption from institutional review board review (IRB No. E2509/003-010). Data access and analysis were conducted in a secure data environment managed by the Korea Foundation for Suicide Prevention. Export of raw data was restricted, and only approved statistical output files were released for research use.

## 3 Results

### 3.1 Presence of suicide warning signs across age groups

Among 96,501 suicide decedents with valid data on age group and warning sign status, 89,668 (92.9%) had at least one suicide warning sign. As described in the Methods, this variable was derived from standardized coding of police investigation reports and should be interpreted within the record-based ascertainment process. The proportion of decedents with warning signs differed significantly across age groups [*χ*^2^(3) = 433.202, *p* < 0.001]. Warning signs were most frequently observed among older adults (95.1%), followed by middle-aged adults (92.9%), young adults (90.9%), and children/adolescents (87.4%) (Table 1).

**Table 1:** Presence of suicide warning signs by age group.

Age group	No warning sign (n)	Warning sign (n)	Total (N)	Warning sign (%)
Children/adolescents	259	1,797	2,056	87.4
Young adults	2,045	20,356	22,401	90.9
Middle-aged adults	3,242	42,402	45,644	92.9
Older adults	1,287	25,113	26,400	95.1
Total	6,833	89,668	96,501	92.9

In the adjusted logistic regression model including age group and sex, all age groups younger than older adults had significantly lower odds of having any warning sign. Compared with older adults, the odds were lowest among children/adolescents (OR = 0.331, 95% CI [0.287, 0.382], *p* < 0.001), followed by young adults (OR = 0.495, 95% CI [0.460, 0.532], *p* < 0.001) and middle-aged adults (OR = 0.688, 95% CI [0.643, 0.735], *p* < 0.001). Because the outcome was common, these ORs should be interpreted alongside the observed absolute proportions shown in Table 1. Male decedents also had lower odds of having any warning sign than female decedents (OR = 0.583, 95% CI [0.550, 0.619], *p* < 0.001). The model was statistically significant [*χ*^2^(4) = 765.364, *p* < 0.001], with Nagelkerke *R*^2^ = 0.020 (Table 2).

**Table 2:** Logistic regression for the presence of any suicide warning sign.

Predictor	*B*	SE	OR	95% CI	*p*
Male	−0.539	0.031	0.583	0.550–0.619	<0.001
Children/adolescents	−1.105	0.073	0.331	0.287–0.382	<0.001
Young adults	−0.704	0.037	0.495	0.460–0.532	<0.001
Middle-aged adults	−0.374	0.034	0.688	0.643–0.735	<0.001

Reference groups were female for sex and older adults for age group. Model chi-square (4) = 765.364, *p* < 0.001; Nagelkerke *R*^2^ = 0.020.

### 3.2 Distribution of warning sign domains by age group

Table 3 shows the composition of warning sign occurrences across age groups. Because the denominator was the total number of warning sign occurrences within each age group, these percentages do not represent person-level domain prevalence. Verbal warning signs accounted for the largest share of warning sign occurrences among older adults (46.6%), whereas emotional warning signs accounted for the largest share among young adults (40.0%) and children/adolescents (39.4%). Middle-aged adults showed a more balanced composition between verbal warning signs (36.6%) and emotional warning signs (36.0%). Behavioral warning signs accounted for similar shares among young adults (27.8%), children/adolescents (27.7%), and middle-aged adults (27.4%), but a smaller share among older adults (19.0%).

**Table 3:** Composition of warning sign occurrences by age group.

Age group	Verbal, *n* (%)	Behavioral, *n* (%)	Emotional, *n* (%)	Total warning sign occurrences
Children/adolescents	897 (32.9)	755 (27.7)	1,072 (39.4)	2,724
Young adults	10,115 (32.2)	8,725 (27.8)	12,582 (40.0)	31,422
Middle-aged adults	25,244 (36.6)	18,868 (27.4)	24,789 (36.0)	68,901
Older adults	19,606 (46.6)	7,978 (19.0)	14,515 (34.5)	42,099

Percentages are based on the total number of warning sign occurrences within each age group, not on the number of decedents. Warning sign domains were not mutually exclusive; therefore, percentages describe the composition of warning sign occurrences and should not be interpreted as person-level domain prevalence.

### 3.3 Age-specific patterns in individual warning signs

Item-level logistic regression analyses showed distinct age-specific patterns across verbal, behavioral, and emotional warning signs (Table 4). These models were age-group-only descriptive models. Compared with older adults, children/adolescents, young adults, and middle-aged adults all had lower odds of verbal warning signs, including suicide-related expressions, complaints of physical discomfort, and self-deprecating statements. The largest age-group differences were observed for complaints of physical discomfort, with markedly lower odds among children/adolescents (OR = 0.099, 95% CI [0.081, 0.120], *p* < 0.001), young adults (OR = 0.133, 95% CI [0.125, 0.141], *p* < 0.001), and middle-aged adults (OR = 0.305, 95% CI [0.294, 0.316], *p* < 0.001).

**Table 4:** Age-group differences in specific suicide warning signs.

Domain/warning sign	Children/adolescents OR (95% CI)	Young adults OR (95% CI)	Middle-aged adults OR (95% CI)	Nagelkerke *R*^2^
Verbal: suicide-related expressions	0.449 (0.406–0.496)^***^	0.424 (0.408–0.441)^***^	0.568 (0.549–0.588)^***^	0.032
Verbal: physical discomfort	0.099 (0.081–0.120)^***^	0.133 (0.125–0.141)^***^	0.305 (0.294–0.316)^***^	0.134
Verbal: self-deprecating statements	0.787 (0.638–0.971)^*^	0.875 (0.811–0.944)^**^	0.821 (0.771–0.875)^***^	0.001
Behavioral: indifference to appearance management	1.487 (0.680–3.253)	2.223 (1.636–3.022)^***^	2.375 (1.803–3.129)^***^	0.009
Behavioral: self-harm/suicide attempt/substance misuse	1.428 (1.280–1.593)^***^	1.569 (1.502–1.639)^***^	1.875 (1.806–1.946)^***^	0.020
Behavioral: apathy/interpersonal withdrawal/loss of interest	1.403 (1.234–1.596)^***^	1.330 (1.261–1.402)^***^	1.197 (1.143–1.254)^***^	0.003
Emotional: anxiety	1.910 (1.665–2.190)^***^	1.904 (1.796–2.019)^***^	1.511 (1.433–1.593)^***^	0.012
Emotional: depressed mood	0.931 (0.835–1.037)	1.058 (1.014–1.104)^**^	0.979 (0.944–1.014)	0.000
Emotional: elevated mood	2.830 (2.178–3.677)^***^	2.580 (2.268–2.935)^***^	1.754 (1.552–1.983)^***^	0.014

Older adults were the reference group. Item-level models included age group only and were not adjusted for sex. Analytic *N* varied by warning sign item: verbal models, 82,795–82,813; behavioral models, 76,912–76,918; emotional models, 76,232–76,255. ^*^p < 0.05, ^**^p < 0.01, ***p < 0.001.

Behavioral warning signs showed the opposite pattern for several items. Compared with older adults, children/adolescents, young adults, and middle-aged adults had higher odds of self-harm, suicide attempts, or substance misuse, as well as apathy, interpersonal withdrawal, or loss of interest. Indifference to appearance management was significantly more likely among young adults (OR = 2.223, 95% CI [1.636, 3.022], *p* < 0.001) and middle-aged adults (OR = 2.375, 95% CI [1.803, 3.129], *p* < 0.001), but the difference was not statistically significant among adolescents (OR = 1.487, 95% CI [0.680, 3.253]).

For emotional warning signs, anxiety and elevated mood were more likely in children/adolescents, young adults, and middle-aged adults than in older adults. Anxiety showed similar elevations among children/adolescents (OR = 1.910, 95% CI [1.665, 2.190], *p* < 0.001) and young adults (OR = 1.904, 95% CI [1.796, 2.019], *p* < 0.001), with a smaller but still significant elevation among middle-aged adults (OR = 1.511, 95% CI [1.433, 1.593], *p* < 0.001). Elevated mood was also more likely in all three younger age groups, with the highest odds among children/adolescents (OR = 2.830, 95% CI [2.178, 3.677], *p* < 0.001), followed by young adults (OR = 2.580, 95% CI [2.268, 2.935], *p* < 0.001) and middle-aged adults (OR = 1.754, 95% CI [1.552, 1.983], *p* < 0.001). Depressed mood showed a more limited age-group pattern: young adults had slightly higher odds than older adults (OR = 1.058, 95% CI [1.014, 1.104], *p* < 0.01), whereas adolescents and middle-aged adults did not differ significantly from older adults.

## 4 Discussion

This study examined age-specific profiles of suicide warning signs among Korean suicide decedents using nationwide police-record-based data from 2013 to 2020. Warning signs were common among suicide decedents, but their profiles differed meaningfully by age group. Older adults had the highest proportion of any warning sign and showed a profile characterized by verbal signs, particularly complaints of physical discomfort. In contrast, children/adolescents and young adults had lower overall proportions of warning signs, but emotional warning signs accounted for the largest share of warning sign occurrences in these groups. Behavioral warning signs were also more pronounced among children/adolescents, young adults, and middle-aged adults than among older adults for several items. These findings suggest that suicide warning signs should be understood as age-specific profiles rather than as a uniform set of indicators.

The high proportion of warning signs across all age groups is consistent with prior work suggesting that observable changes are often reported before suicide (7). However, the present findings add that the type of warning sign may be as important as its presence. Warning signs are conceptually closer to acute suicide risk than distal risk factors, and their preventive value depends on whether they can be recognized by people around the individual before death (4). At the same time, the present study cannot determine whether recognition of these signs would have prevented suicide, because the data included suicide decedents only and did not include suicide attempt survivors or a non-suicide comparison group. Therefore, the findings should be interpreted as evidence that may inform future prevention strategies and surveillance systems, rather than as direct evidence of preventive effectiveness.

Older adults showed the clearest profile of verbal warning signs, particularly complaints of physical discomfort. They had the highest overall proportion of warning signs, and all younger age groups had lower odds of verbal warning signs compared with older adults. This pattern may reflect late-life contexts in which physical illness, pain, functional decline, bereavement, loneliness, and loss of independence are closely tied to suicide risk (10, 15, 16). An autopsy-based study from Türkiye similarly reported that many suicide decedents had systemic disease, psychiatric disorders or psychiatric medication use, or both, underscoring the relevance of medical and psychiatric history when interpreting suicide deaths (17). Prior studies of older suicide decedents have also shown that physical health problems are common precipitants and may be associated with depressed mood, suicidal intent disclosure, pain, cancer, functional decline, fear of becoming a burden, and loss of independence (10, 15). Therefore, for older adults, families, caregivers, primary care providers, and community health workers should attend not only to direct suicidal statements but also to repeated physical complaints, expressions of burden, withdrawal in the context of illness, and communication about death or life fatigue.

Children/adolescents and young adults showed a different profile. Although their overall proportion of warning signs was lower than that of older adults, emotional warning signs accounted for the largest proportion of warning sign occurrences in both groups. This finding is important because emotional warning signs may be less explicit than direct suicidal communication and may be interpreted as ordinary distress, developmental turbulence, or interpersonal conflict. Korean psychological autopsy research has shown that warning signs among adolescents may be difficult to recognize when emotional distress is concealed or when school and peer functioning appears outwardly preserved (8). Meta-analytic evidence also suggests that adolescent anxiety is associated with subsequent suicidal behavior (12). Recent national data from Türkiye further emphasize that suicide deaths vary by age, sex, geography, life circumstances, and known reasons such as illness and economic problems, supporting the broader view that suicidal behavior is shaped by psychosocial and contextual life experiences rather than by a single clinical pathway (18). These findings support the need for families, peers, teachers, school personnel, university staff, and youth-serving gatekeepers to recognize anxiety, abrupt mood changes, emotional volatility, withdrawal, and self-harm history as potentially meaningful warning signs, even when explicit suicidal statements are absent.

The emotional warning sign findings also caution against equating suicide warning signs only with depressed mood or verbalized suicidal intent. In this study, anxiety and elevated mood were more likely among children/adolescents, young adults, and middle-aged adults than among older adults, whereas depressed mood was only slightly higher among young adults and did not differ significantly among children/adolescents or middle-aged adults. This pattern suggests that emotional warning signs are heterogeneous and may involve anxiety, agitation, mood instability, or other forms of affective dysregulation, not only depressive presentation. This interpretation is consistent with broader models of suicidal behavior that emphasize dynamic interactions among psychological pain, entrapment, impulsivity, capability, and volitional factors rather than a single emotional pathway (2, 19). Gatekeeper education should therefore include a broader emotional vocabulary for recognizing suicide risk, particularly in younger groups where distress may be intense but not directly disclosed.

The sex finding in the primary model also warrants brief attention. Male decedents had lower odds of having any warning sign than female decedents, despite the higher burden of suicide mortality among men in many settings. This pattern may reflect sex differences in help-seeking, disclosure of distress, interpersonal communication, or the likelihood that others recognize warning signs before death. From a prevention perspective, this finding underscores the need to train families, peers, workplaces, and community gatekeepers to recognize less verbal, less help-seeking, or more behaviorally expressed forms of distress among men.

Behavioral warning signs further differentiated the age groups. Children/adolescents, young adults, and middle-aged adults had higher odds than older adults of self-harm, suicide attempts, or substance misuse, as well as apathy, interpersonal withdrawal, or loss of interest. These findings suggest that in non-older age groups, suicide risk may become visible through actions, disengagement, and functional decline rather than primarily through verbal expressions. Prior reviews have identified self-harm, substance misuse, psychiatric problems, and social or occupational stressors as important correlates of suicidality across adolescence, adulthood, and midlife (1, 13, 20, 21). For middle-aged adults in particular, behavioral changes may need to be interpreted in relation to employment, financial strain, family responsibilities, relationship disruption, and health problems (13, 21). Workplace supervisors, family members, community workers, and gatekeepers should therefore regard sudden withdrawal, loss of interest, substance-related changes, and deterioration in daily functioning as potentially meaningful warning signs.

A notable behavioral finding was that indifference to appearance management was significantly more likely among young adults and middle-aged adults than among older adults, but not significantly different among adolescents. This pattern may reflect the social visibility of appearance and self-care in young and middle adulthood, when daily life is often structured around school, work, romantic relationships, family roles, and public-facing social expectations. Neglect of appearance in these age groups may therefore be more readily noticed as a change from usual functioning or as a marker of withdrawal, apathy, or reduced self-care. In contrast, the non-significant finding among adolescents should not be interpreted as evidence that appearance-related changes are unimportant. It may reflect lower statistical precision, developmental variation in appearance norms, or the possibility that adolescent distress is more likely to be concealed or interpreted through school and peer contexts rather than identified as appearance neglect (8). Thus, appearance-related changes should be interpreted contextually and alongside other emotional or behavioral signs.

Taken together, these findings indicate that the public health value of warning sign research depends less on whether any warning sign was present than on which warning signs appeared in each age group. Older adults showed the highest overall proportion of warning signs, but these signs were more often verbal, particularly complaints of physical discomfort. Children/adolescents and young adults showed lower overall proportions of warning signs, yet their profiles were more strongly characterized by emotional and behavioral warning sign occurrences. Middle-aged adults showed a mixed profile, with verbal and emotional warning sign distributions appearing relatively balanced and several behavioral warning signs elevated compared with older adults. This distinction suggests that suicide prevention should move beyond a universal checklist and teach families, peers, community members, clinicians, workplace personnel, school staff, and gatekeepers what warning signs may look like at different stages of life.

Several limitations should be considered. First, this study used retrospective secondary data derived from police investigation records. Although the data were coded by trained investigators using a standardized record-based framework developed by the Korea Foundation for Suicide Prevention, warning signs were identified from information available in police investigation reports and may still have been underreported or differentially ascertained by age group. Ascertainment may also have depended on the availability of informants, the decedent’s social contact, and the circumstances of death discovery; warning signs may have been more readily identified when family members, coworkers, or other informants were available. Second, the data did not establish the timing, duration, or sequence of warning signs before death, although prior psychological autopsy work suggests that warning signs may vary by proximity to death (6). Third, warning sign domains were not mutually exclusive, and percentages based on warning sign occurrences should not be interpreted as mutually exclusive person-level prevalence. Fourth, item-level logistic regression models used older adults as the reference group and did not adjust for sex because they were intended to describe age-specific warning sign profiles. The estimates therefore describe descriptive age-group differences relative to older adults rather than fully adjusted or causal associations. Fifth, because the outcome of any recorded warning sign was common, ORs may appear larger than corresponding differences in absolute proportions and should be interpreted alongside the observed proportions. Finally, the study pooled deaths from 2013 to 2020, and secular changes across this period, including possible changes in social contact, help-seeking, and documentation during the first year of the COVID-19 pandemic, may have influenced the observed patterns. Despite these limitations, this study provides nationwide evidence that documented suicide warning signs differ by age group among Korean suicide decedents. Future suicide mortality surveillance would be strengthened by systematically recording the source of warning sign information, informant availability, timing of warning signs, healthcare contact, psychiatric history, social isolation, and relevant recent life events.

## 5 Conclusion

This study found that suicide warning signs were common among Korean suicide decedents, but their profiles differed by age group. Older adults showed the highest overall proportion of warning signs, with a profile characterized more by verbal signs, particularly complaints of physical discomfort. In contrast, children/adolescents and young adults had lower overall proportions of warning signs, but emotional and behavioral warning sign occurrences accounted for a larger share of their profiles. Middle-aged adults showed a mixed profile, with relatively balanced verbal and emotional warning sign distributions and elevated behavioral warning signs compared with older adults.

These findings suggest that future suicide prevention efforts should move beyond a uniform, age-neutral checklist of warning signs. The key issue is not only whether warning signs are present, but which signs are most likely to become visible at different stages of life. Age-sensitive recognition strategies may help families, friends, community members, healthcare providers, school personnel, workplace supervisors, and gatekeepers recognize warning signs that are less explicit, less verbal, or more context-specific. Because this study included only suicide decedents, these findings should be viewed as informing future prevention strategies rather than demonstrating preventive effectiveness.

## Data Availability

The datasets presented in this article are not readily available because restrictions apply to the availability of these data, which were used under license for the present study and are therefore not publicly available. Raw data could not be exported outside the secure analysis environment; only approved statistical output files were released for research use. Requests to access the datasets should be directed to Jiu Kim, wldn825@dongguk.ac.kr.
